# The Entomopathogenic Fungi *Isaria fumosorosea* Plays a Vital Role in Suppressing the Immune System of *Plutella xylostella*: RNA-Seq and DGE Analysis of Immunity-Related Genes

**DOI:** 10.3389/fmicb.2017.01421

**Published:** 2017-07-28

**Authors:** Jin Xu, Xiaoxia Xu, Muhammad Shakeel, Shuzhong Li, Shuang Wang, Xianqiang Zhou, Jialin Yu, Xiaojing Xu, Xiaoqiang Yu, Fengliang Jin

**Affiliations:** ^1^Laboratory of Bio-Pesticide and Application of Guangdong Province, College of Agriculture, South China Agricultural University Guangzhou, China; ^2^Beijing Genomic Institute Shenzhen, China; ^3^School of Biological Sciences, University of Missouri-Kansas Kansas City, MO, United States

**Keywords:** *Plutella xylostella*, RNA-Seq, *Isaria fumosorosea*, immune genes, DGE

## Abstract

Most, if not all, entomopathogenic fungi have been used as alternative control agents to decrease the insect resistance and harmful effects of the insecticides on the environment. Among them, *Isaria fumosorosea* has also shown great potential to control different insect pests. In the present study, we explored the immune response of *P. xylostella* to the infection of *I. fumosorosea* at different time points by using RNA-Sequencing and differential gene expression technology at the genomic level. To gain insight into the host-pathogen interaction at the genomic level, five libraries of *P. xylostella* larvae at 12, 18, 24, and 36 h post-infection and a control were constructed. In total, 161 immunity-related genes were identified and grouped into four categories; immune recognition families, toll and Imd pathway, melanization, and antimicrobial peptides (AMPs). The results of differentially expressed immunity-related genes depicted that 15, 13, 53, and 14 up-regulated and 38, 51, 56, and 49 were down-regulated in *P. xylostella* at 12, 18, 24, and 36 h post-treatment, respectively. RNA-Seq results of immunity-related genes revealed that the expression of AMPs was reduced after treatment with *I. fumosorosea*. To validate RNA-Seq results by RT-qPCR, 22 immunity-related genes were randomly selected. In conclusion, our results demonstrate that *I. fumosorosea* has the potential to suppress the immune response of *P. xylostella* and can become a potential biopesticide for controlling *P. xylostella*.

## Introduction

Insects are surrounded by an environment rich with harmful microorganisms and recurring infections are common in the natural environment. In order to combat these potentially infectious pathogens, insects have evolved various defense systems, including the potent immune system. Unlike mammals, insects solely rely on innate immune responses for host defense. The innate immune responses are usually comprised of cellular and humoral defense responses. The former is best demonstrated by the action of hemocytes in the phagocytosis (Kanost et al., 2004) whereas the hallmark of latter is the synthesis of antimicrobial peptides (AMPs) (Hoffmann and Reichhart, 2002). Upon microbial infection, cellular, and humoral responses are activated by insects, to clear the infection, through different steps (Söderhäll and Cerenius, 1998). The invading pathogen is recognized via pattern recognition receptors (PRRs) (Hultmark, 2003) leading to the amplification of signal of infection by serine proteases following the activation of signaling pathways (Jiang and Kanost, 2000; Osta et al., 2004). Finally, the effector factors are induced in the specific tissues to combat the pathogens.

To counter the defense system of the host, insect pathogenic fungi have also developed their mechanisms. The pathogens use a set of enzymes to breach the cuticle (Butt, 2002) and also release secondary metabolites, to suppress the immune system of the host, during colonization (Vilcinskas et al., 1997; Vey et al., 2002). Among these entomopathogenic fungi, on one hand, *Metarhizium anisopliae* has developed a new technique to evade the immune system of host via masking the cell wall during hemocoel colonization (Wang and Leger, 2006), and on the other hand, *Isaria fumosorosea releases* chitinase, chitosanase, lipase, to physically penetrate the host and suppress its regulatory system, and a beauvericin compound to paralyze the host (Hajek and St. Leger, 1994; Ali et al., 2010).

The diamondback moth (DBM), *Plutella xylostella* (L.) (Lepidoptera: Plutellidae), is one of the devastating pests of brassicaceous crops and costs approximately US$4-5 billion per year worldwide (Zalucki et al., 2012). *P. xylostella* is commonly known to rapidly evolve resistance against almost all types of insecticides including products of *Bacillus thuringiensis* (Shakeel et al., 2017). Consequently, entomopathogenic fungi have received an increased attention as an environmentally friendly alternative control measure to insecticides for controlling *P. xylostella*. Several strains of fungi have been isolated and used to control various insect pests including *P. xylostella* (Altre et al., 1999; Leemon and Jonsson, 2008; Bukhari et al., 2011). Of these entomopathogenic fungi, *I. fumosorosea* is considered as one of the promising species of fungi to be used as biological control of insect pests and various mycopesticide based on *I. fumosorosea* have been developed worldwide (Zimmermann, 2008). *Isaria fumosorosea*, a well-known entomopathogenic fungi, is distributed worldwide. It was previously known as *Paecilomyces fumosoroseus*, however, now it has been transferred to Isaria genus (Zimmermann, 2008). Due to wide host range, it has become a promising biological control agent and its potential as a biological control agent, other than immunity, has been tested to control various insect pests, including *Diaphorina citri* (Avery et al., 2011), *Bemisia tabaci* (Huang et al., 2010), *Trialeurodes vaporariorum* (Gökçe and Er, 2005), and *P. xylostella* (Huang et al., 2010).

Previously, most of the reports on insect immunity preferred model insects, including *Drosophila melanogaster* (Wraight et al., 2010), *Manduca sexta* (Kanost et al., 2004), and *Tenebrio molitor* (Kim et al., 2008) against insect pathogenic fungi such as *M. acridium* and *Beauveria bassiana* (Xiong et al., 2015; Zhang et al., 2015). It is only recently that *P. xylostella* immunity has received the attention of few researchers, thanks to the availability of the genome sequence of *P. xylostella* (You et al., 2013). Although, a recent report on the immune response of *P. xylostella* to *B. bassiana* improved our information of insect-pathogen interaction (Chu et al., 2016). However, the changes that occur in response to *I. fumosorosea* in *P. xylostella* are largely unclear, restricting the development of fungal species other than *M. anisopliae* and *B. bassiana* to be adopted as a biological control agent in *P. xylostella* and other lepidopteran pests' control.

To gain deep insight into the immunogenetics of *P. xylostella*, the present study conducted a genome-wide profiling analysis of *I. fumosorosea* challenged *P. xylostella* larvae at 12, 18, 24, and 36 h post-infection using RNA-Seq and digital gene expression (DGE). Additionally, a global survey of the activities of anti-fungal immune defense genes in *P. xylostella* may also contribute to the in-depth analysis of candidate genes in *P. xylostella* immunity.

## Materials and methods

### Insect stock

The population of susceptible *P. xylostella* was kindly provided by Institute of Plant Protection, Guangdong Academy of Agricultural Sciences, China and was maintained in the Engineering Research Centre of Biological Control Ministry of Education, South China Agricultural University, Guangzhou, Guangdong province, P. R. China for five years without exposure to pesticides. Larvae were maintained at 25 ± 1°C with a light: dark cycle of 16:8 h and 60–70% relative humidity.

### Fungus culture, conidia suspension preparation, and samples collection

The *I.fumosorosea* IfB01 strain (China Center for Type Culture Collection access number: CCTCC M 2012400) was cultured on a potato dextrose agar (PDA) plate at 26°C. The conidia were collected from 10 days old culture and suspended with 0.05% Tween-80 into standardized 1 × 10^8^ spores/mL (Huang et al., 2010). Healthy *P. xylostella* larvae (third-instar) were selected and separated into two groups. One group (treatment) was treated with the 1 × 10^7^ spores/ mL suspension, whereas the other group (control) was treated with sterile deionized water containing 0.05% Tween-80. The samples of 50 surviving larvae were collected from the treatment group and the control group at 12, 18, 24, and 36 h, respectively, forming three pairs of hour post-treatment infection and hours post treatment control. Different time-points of sampling were selected to observe infection dynamics (Abkallo et al., 2015) and dynamical changes (Bar-Joseph et al., 2012) of differentially expressed genes (DEGs) in response to *Isaria fumosorosea* in *Plutella xylostella*, as the gene expression profiling of different time points can provide DEGs dynamical behavior information.

### Preparation of cDNA library and illumina sequencing

A total of five DGE libraries (12, 18, 24, 36 h, and control) were produced by the Illumina Gene Expression Sample Prep Kit (Illumina, San Diego, CA). Total RNA (10 μg) was isolated from each treatment and control for the isolation of poly (A)^+^ mRNA using oligo (dT) magnetic beads. cDNAs (First- and second-strand) were prepared using random hexamers, RNase H, and DNA polymerase I. Magnetic beads were used to purify the double strand cDNA and finally, ligation of fragments was carried out with sequencing adaptors. To quantify and qualify the libraries of samples, Agilent 2100 Bioanalyzer and ABI Step One Plus Real-Time PCR System were employed and then sequencing was done on the Illumina HiSeq™ 2000 system (Illumina, USA). Illumina sequencing was carried out at the Beijing Genomics Institute (BGI-Shenzhen, China).

### Mapping and functional analysis of differentially expressed genes

The process of filtration was performed in such a way that raw reads with adopters and unknown bases (more than 10%) were removed. After filtering, the remaining clean reads were mapped to reference gene using Bowtie (Langmead et al., 2009) and HISAT (Kim et al., 2015) was employed to map the reference genome. Finally, normalization of all data was done as fragments per kilobase of transcript per million fragments mapped (FPKM). The analysis of differential expression was employed by a rigorous algorithm. The false discovery rate (FDR) methodology was adopted in multiple tests (Kim and van de Wiel, 2008) for determination of threshold of *P*-value. Genes with significant differential expression were searched out according to a standard threshold having an FDR value of < 0.001 and the absolute value of log2 ratio ≥ 1. The genome database of *P. xylostella* was used as the background to determine significantly enriched GO terms and Kyoto Encyclopedia of Genes and Genomes (KEGG) pathway enriched within the DEG dataset using hypergeometric test and a corrected *P*-value (≤0.05) as a threshold.

### Validation of DEGs libraries by RT-qPCR

In order to validate mRNA expression levels exhibited by RNA-Seq results, Real-time quantitative PCR (RT-qPCR) was performed with 22 immunity-related DEGs chosen from the comparison of control vs. treatments. Total RNA isolation method was same as described earlier. In total, 1 μg of total RNA was treated with DNaseI (Fermentas, Glen Burnie, MD, USA) according to the instructions of the manufacturer and then cDNA was prepared using M-MLV reverse transcriptase (Promega, USA). The RT-qPCR was performed on a Bio-Rad iQ2 optical system (Bi-Rad) using SsoFast EvaGreen Supermix (Bio-Rad, Hercules, CA, USA) according to guidelines provided by the manufacturer. To confirm the PCR products purity, the amplification cycling parameters were set as; 95°C for 30 s, 40 cycles of 95°C for 5 s, and 55°C for 10 s with a dissociation curve generated from 65 to 95°C (Shakeel et al., 2015). For normalization, ribosomal protein S13 (RPS13) was used as an internal control (Fu et al., 2013) and the relative expression of genes was calculated using the 2^−ΔΔ^^CT^ method (Livak and Schmittgen, 2001). The primers used for RT-qPCR are listed in Table 1.

**Table 1:** Primers used for RT-qPCR in the present study.

**Gene name**	**Gene ID**	**Direction**	**Sequence (5′–3′)**
Px_Tryp_SPN12	105393249	Forward	GCAGACCTTGGTTATATC
		Reverse	GATGAAGCTCTTGTACTC
Px_ChymTryp_SP6	105397690	Forward	GAAGTGTTCTGATTGGAG
		Reverse	TAGATACGAGCGTTGATC
Px_PPO1	105393828	Forward	GATCAAGCCTAAGGTATG
		Reverse	GTCACCATCTTCTGTATC
Px_Catalase1	105398438	Forward	CCGTTTTCTACACTAAGG
		Reverse	GGTACTTCTTGTAAGGAG
Px_Lectin2	105395555	Forward	GAGACAGTTTAGTTCCCT
		Reverse	GAAGTAGCCCTTGTTATC
Px_SP20	105380853	Forward	GCTATGTTGTGCATACAG
		Reverse	CATATTCTGCGAGTAGTC
Px_PGRP1	105387866	Forward	GTATAATTTCTGCGTGGG
		Reverse	CTCCAATCTCCAATAAGAC
Px_Lectin6	105392913	Forward	GATCAAGAGGATGGTTAC
		Reverse	CTTCAGTTCCCTTCTATC
Px_Moricin1	105392531	Forward	ATGAGATTCCTCCACTTG
		Reverse	CCTTCCGAATAACTCTTC
Px_Serpin1	105396587	Forward	GACTCGGAGGATATTTAC
		Reverse	CCAGGTCTAAGATGTATTG
Px_βGBP1	105380182	Forward	GGAAAGGATACCTGAAAG
		Reverse	GAAGTCGTCATAGAAGAC
Px_Tryp_SP1	105381636	Forward	CCAGGAGAAGGATATTCT
		Reverse	CATGATAGAGTCATCCTC
Px_βGBP3	105391537	Forward	CAACTACTACCATGAAGG
		Reverse	GCTCTAGGTTTATCTCAG
Px_Cecropin1	105394859	Forward	CAGGTGGAATCCGTTCAA
		Reverse	GAAGTGGCTTGTCCTATGA
Px_Moricin3	105392532	Forward	GATTCTTCCACTTGCTGATG
		Reverse	CCTTCCGTATAACTCTTCCG
Px_Lectin4	105392416	Forward	CAGGATAAGGTGAAGTACATCT
		Reverse	CCGTCGTTGTAGAAGTTGT
Px_Hemolin1	105394779	Forward	GATTGGTGGAGCAGTATGT
		Reverse	TGGTGTTCTTGATGATGAGT
Px_Peroxidase2	105388497	Forward	CCACCGAGCAACAAGAAT
		Reverse	GAACCATACCGTCATCAGAT
Px_Gloverin2	105389803	Forward	GCCACTCAAGGACATCTT
		Reverse	CTCACTGTTCTTGCCAATC
Px_SCR6	105393261	Forward	GAAGACGGCATCCAACTG
		Reverse	CATAGAACAAGCGGTGACA
Px_SCR7	105394486	Forward	GAAGACGGCATCCAACTG
		Reverse	TAGAGCAAGCGGTGACAT
Px_SP4	105380869	Forward	CTCTGGTGCTATTGCTCTT
		Reverse	GATGGTAGATGTGGTGATGA
RPS13	Reference gene	Forward	TCAGGCTTATTCTCGTCG
		Reverse	GCTGTGCTGGATTCGTAC

## Results and discussion

### Features of the sequenced cDNA libraries

To identify genes involved in *P. xylostella* immunity in response to *I. fumosorosea*, five cDNA libraries were constructed from 3rd larval instar of *P. xylostella* at 12, 18, 24, 36 h after fungal treatment and control. A total of 11,652,857, 11,819,310, 12,051,947, 11,744,46, and 11,683,647 reads were generated from these five libraries (12, 18, 24, 36 h, and control respectively), from which 70.01, 73.55, 73.23, 70.11, and 71.94% reads could be successfully mapped to the reference genome (Table 2).

**Table 2:** DGE sequencing statistics.

**Sample**	**Clean reads**	**Total mapped of clean data (%)**
12 h	11,652,857	70.01
18 h	11,819,310	73.55
24 h	12,051,947	73.23
36 h	11,744,46	70.11
Control	11,683,647	71.94

### Dynamics of differentially expressed immunity-related genes in response to *I. fumosorosea*

**Figure 1:**
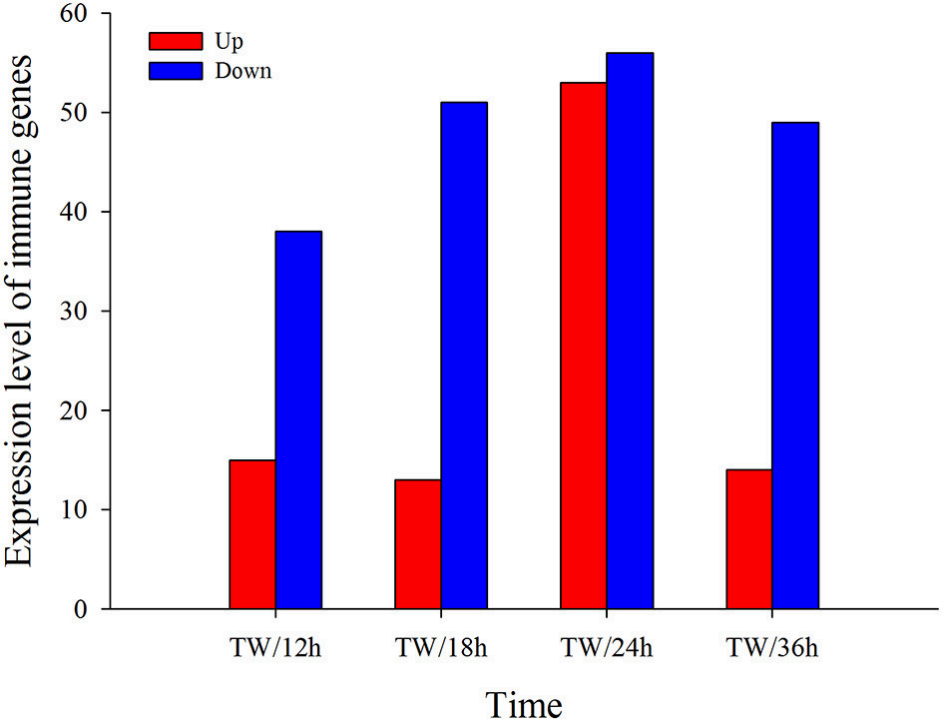
Screening of immunity-related DEGs in response to *I. fumosorosea* at 12, 18, 24, and 36 h post-infection.

**Figure 2:**
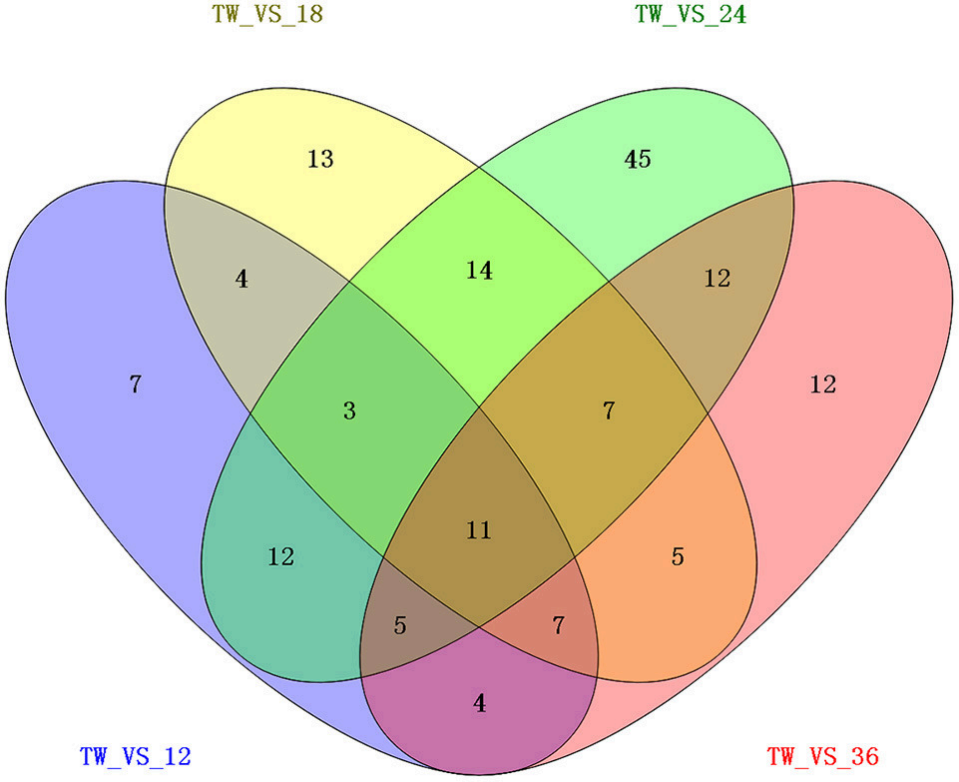
A Venn diagram of differentially expressed immunity-related genes in *P. xylostella* at 12, 18, 24, and 36 h post-infection. The numbers in each circle show differentially expressed genes in each comparison treatment and the overlapping regions display genes that are commonly expressed among the comparison treatments.

To study the gene expression of *P. xylostella* larvae infected with *I. fumosorosea*, the pairwise comparison was carried out between libraries to determine the DEGs. The analysis of five libraries was carried out by determining the number of fragments per kb per million (FPKM) of clean reads. Relative to control, genes with (FDR) ≤ 0.001 and |log2Ratio| ≥ 1 were recognized as differentially expressed. Our results exhibited that, compared to the control, there were 53 (15 up-regulated and 38 down-regulated), 64 (13 up- and 51 down-regulated), 109 (53 up-regulated and 56 down-regulated), and 63 (14 up- and 49 down-regulated) immune-related genes that were significantly changed in *P. xylostella* after 12, 18, 24, and 36 h, respectively (Figure 1). A Venn diagram analysis showed that only 11 immunity-related DEGs were commonly expressed among all the treatments, whereas 7, 13, 45, and 12 immunity-related DEGs were specifically expressed in 12, 18, 24, and 36 h, respectively (Figure 2).

### GO and KEGG classification and enrichment analysis of immunity-related genes in response to *I. fumosorosea*

**Figure 3:**
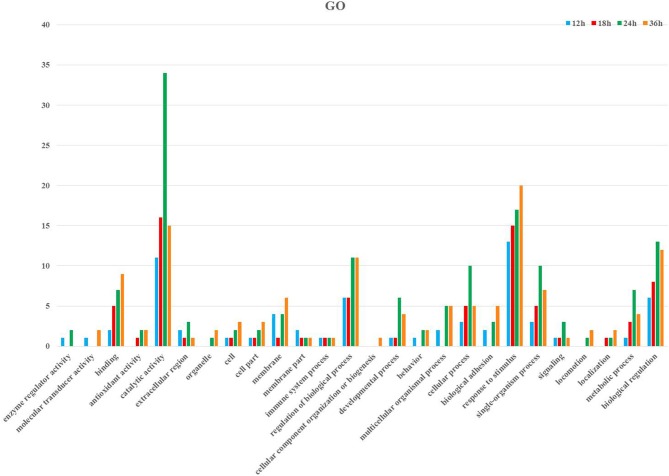
Summary of Gene ontology annotation. Functional classification of immunity- related DEGs at 12, 18, 24, and 36 h post-infection in *P. xylostella* using gene ontology terms.

**Figure 4:**
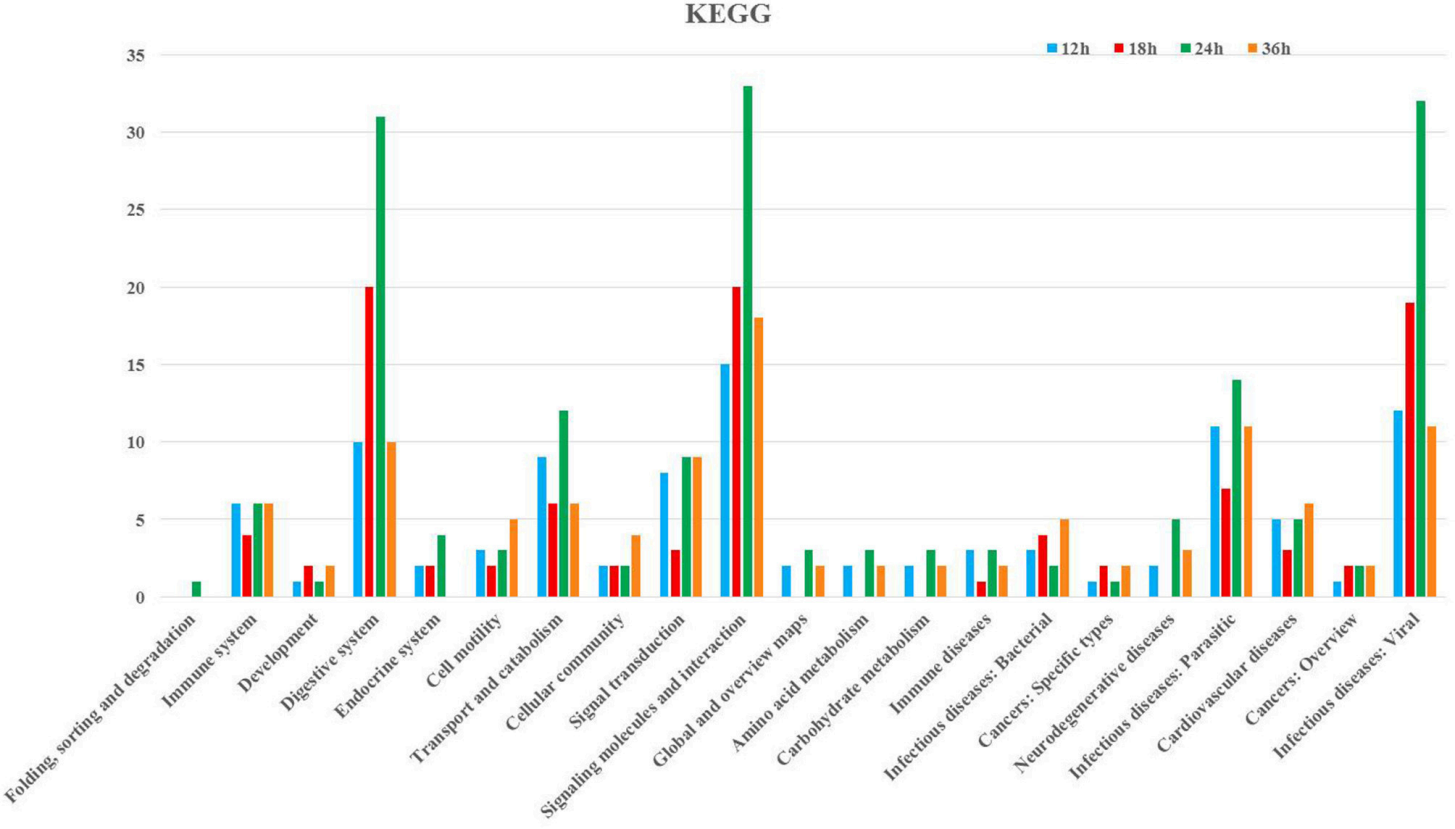
KEGG pathway annotation classification of immunity-related genes in *P. xylostella* infected with *I. fumosorosea* at 12, 18, 24, and 36 h. The abscissa is the KEGG classification, and the ordinate left is the gene number.

Following GO annotation, the immunity-related genes were classified into 26 different groups belonging to biological process, cellular component, and molecular function categories (Figure 3). In the biological process category, the two most enriched groups were the response to stimulus and biological regulation, whereas membrane and regulation of biological process were the top two enriched groups in the cellular component. The number of genes involved in catalytic activity and binding were the dominant groups in the category of molecular function (Figure 3). The KEGG classification system categorized immunity-related genes into 21 different groups (Figure 4). The top five enriched groups among KEGG categories included infectious diseases (viral), signaling molecules and interaction, digestive system, infectious diseases (parasitic), and signal transduction (Figure 4).

### Verification of DEG results by RT-qPCR

**Figure 5:**
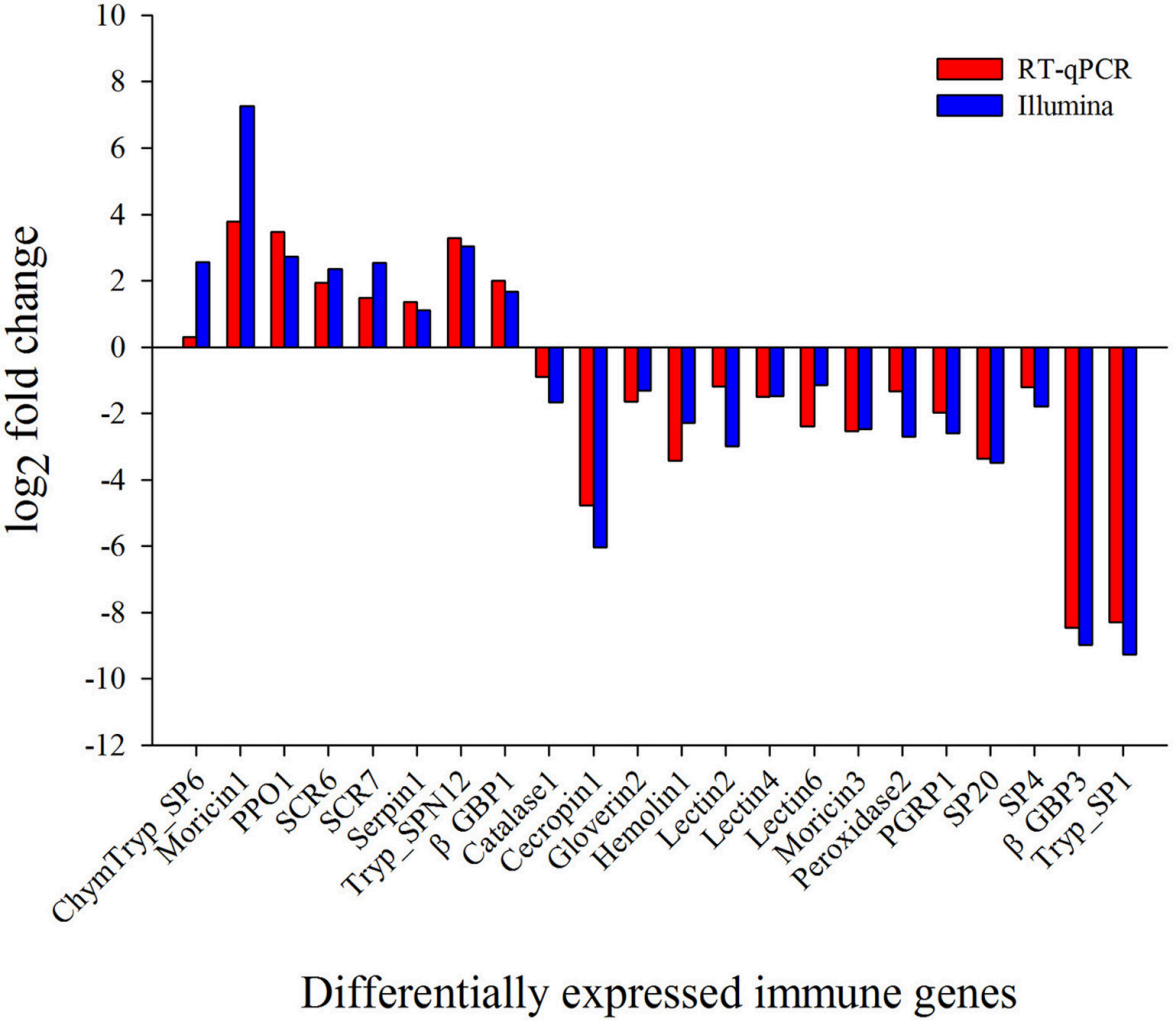
Validation of differential expression ratio (log2) achieved by RT-qPCR and RNA-Seq for immunity-related genes. ChymTryp_SP6, Chymotrypsin like serine protease (Px_105397690); Moricin1, Moricin (Px_105392531); PPO1, prophenoloxidase (Px_105393828); SCR6, Scavenger Receptor (Px_105393261); SCR7, Scavenger Receptor (Px_105394486); Serpin1, Serpin (Px_105396587); Tryp_SPN12, Trypsin-like Serine Protease (Px_105393249); βGBP1, β-1,3-Glucan Binding Protein (Px_105380182); Catalase1, Catalase (Px_105398438); Cecropin1, Cecropin (Px_105394859); Gloverin2, Gloverin (Px_105389803); Hemolin1, Hemolin (Px_105394779); Lectin2, Lectin (Px_105395555); Lectin4, Lectin (Px_105392416); Lectin6, Lectin (Px_105392913); Moricin3, Moricin (Px_105392532); Peroxidase2, Peroxidase (Px_105388497); PGRP1, Peptidoglycan Recognition Protein (Px_105387866); SP20, Serine Protease (Px_105380853); SP4, Serine Protease (Px_105380869); βGBP3, β-1,3-Glucan Binding Protein (Px_105391537); Tryp_SP1, Trypsin-like Serine Protease (Px_105381636).

To validate DEG results, 13 randomly selected immunity-related DEGs were analyzed by RT-qPCR. The results exhibited that the trend of expression level for all the selected genes was in consistence to that of RNA-Seq (Figure 5).

### Identification of immunity-related genes

To identify immunity-related genes in response to *I. fumosorosea*, we searched out the genome of *P. xylostella* and combined BLAST search and GO annotation results. A number of genes having fold change less than one and those annotated as hypothetical or unknown proteins were not selected. Finally, a good number (161) of immunity-related genes were identified and classified as immune recognition families, toll and Imd signaling pathways, melanization, AMPs, and others (Table 3).

**Table 3:** Summary of immunity-related genes identified in *Plutella xylostella* genome.

**Gene name**	**Gene ID**	**Accession no**.	**Gene length**	**Protein length**	**E-value**	**Nr identity**	**Log2**			
							**12 h**	**18 h**	**24 h**	**36 h**
**RECOGNITION**										
**Peptidoglycan recognition protein**										
Px_PGRP1	105387866	AFV15800.1	815	206	2.8223E-60	60.23	−1.4564	−2.6013		−1.6344
Px_PGRP2	105386206	ADU33187.1	1098	211	1.3699E-67	58.71	−1.6343			−1.2606
Px_PGRP3	105387860	ADU33187.1	824	211	3.5191E-66	58.21	−1.7588		1.3324	
Px_PGRP4	105386207	AFV15800.1	761	205	6.5034E-61	60.8		−2.2113	1.1212	
Px_PGRP5	105388663	AFP23116.1	993	193	1.095E-57	59.2		−1.1736		
Px_PGRP6	105391041	BAF36823.1	690	195	4.9765E-91	87.1		−1.4843		−1.0507
Px_PGRP7	105391791	BAF36823.1	863	186	7.9578E-64	64.57			−1.4168	−1.7257
**β-1,3-Glucan binding protein**										
Px_βGBP1	105380182	AHD25001.1	1424	473	6.084E-125	50.22			1.6692	
Px_βGBP2	105394612	Q8MU95.1	1582	482	1.239E-121	46.43	−3.1341	−4.8773	1.1633	−2.7570
Px_βGBP3	105391537	Q8MU95.1	1589	482	3.502E-124	46.53	−2.4046	−8.9744		−2.1931
Px_βGBP4	105390013	Q8MU95.1	1467	481	1.326E-122	48.51		1.0306		
Px_βGBP5	105389999	Q8MU95.1	1577	490	0	65.91		−1.2506		1.1221
Px_βGBP6	105380252	Q8MU95.1	2875	930	9.183E-111	43.64			1.0275	−1.9780
Px_βGBP7	105391544	Q8MU95.1	765	254	9.5958E-44	40.55				5.4919
Px_βGBP8	105397355	Q8MU95.1	1429	428	0	66.95				1.6163
Px_βGBP9	105388931	AFC35297.1	1494	426	2.159E-112	45.23	−5.3923		−5.3923	−5.3923
Px_βGBP10	105388956	AFC35297.1	1098	306	2.5257E-29	44.58	−8.8948	−2.6469	1.2345	−8.8948
Px_βGBP11	105390015	AGT95925.1	755	244	7.8321E-51	45			1.6273	
Px_βGBP12	105394615	AFC35297.1	1391	426	6.98E-110	46.05			−5.7549	
Px_βGBP13	105388955	NP_001128672.1	2895	922	2.792E-107	42.22	2.3049		−4.0875	
Px_βGBP14	105391545	NP_001128672.1	2967	758	2.9017E-89	50	−4.9069		−4.9069	1.0238
Px_βGBP15	105394614	NP_001128672.1	1476	491	2.714E-99	42		−4.9542	1.3496	1.9527
Px_βGBP16	105394613	NP_001128672.1	1153	358	1.5059E-96	44.83				−7.0334
**Scavenger receptor**										
Px_SCR1	105381120	EHJ69946.1	688	229	8.4588E-67	52.21		2.0233		
Px_SCR2	105394003	NP_001164650.1	1,426	369	3.325E-147	64.96			1.8605	
Px_SCR3	105394000	NP_001164651.1	3,147	495	7.71E-151	52.2			1.3314	
Px_SCR4	105392382	XP_004930787.1	2,049	577	0	62.17	2.2657			
Px_SCR5	105393137	XP_004930826.1	2148	633	0	72.76	−1.6092	−2.0639	1.3051	
Px_SCR6	105393261	XP_004930796.1	2,478	571	1.336E-179	54.48	1.2530		2.3480	
Px_SCR7	105394486	XP_004930796.1	1,778	461	9.582E-150	55.73	2.3282		2.5486	
Px_SCR8	105389099	XP_004930796.1	1,922	461	9.814E-172	55	1.2243		2.7444	
Px_SCR9	105383111	EHJ75193.1	2,421	512	6.793E-128	45.65			−1.4056	
**Lectin**										
Px_Lectin1	105383612	BAM17981.1	1,372	293	2.0017E-94	86.32	−1.1543	−1.6011		−1.1441
Px_Lectin2	105395555	BAM17857.1	4,54	123	2.6181E-42	83.33		−2.9970	2.0541	
Px_Lectin3	105382435	AFM52345.1	1,271	223	8.835E-125	93.27				−1.0849
Px_Lectin4	105392416	NP_001091747.1	1,268	223	6.618E-115	95.26			2.9364	−1.4820
Px_Lectin5	105398492	NP_001165397.1	1,156	220	2.173E-111	96.3	−3.5082	−2.6126	1.7611	−2.3971
Px_Lectin6	105392913	EHJ77925.1	1,870	578	1.697E-112	43.03		−1.1576		
Px_Lectin7	105398161	EHJ77925.1	1,810	578	8.125E-112	43.03				1.1921
Px_Lectin8	105383689	AFC35299.1	1,290	307	7.979E-89	52.12		−1.8414		1.2602
**MODULATION**										
**Serine protease**										
Px_SP1	105394363	ADT80832.1	688	200	4.247E-26	37.5	1.2497	1.0804	1.1609	−1.7919
Px_SP2	105381934	AGR92345.1	1,091	270	1.6486E-73	55.74	−1.1914			−3.4241
Px_SP3	105380905	AGR92345.1	2,407	785	2.459E-138	93.33	−1.4117		−2.4052	
Px_SP4	105380869	AGR92345.1	827	252	1.8037E-94	68.07	−2.1802	−2.8343	−4.0062	−1.7866
Px_SP5	105393891	AGR92347.1	275	69	1.6748E-12	68.63		10.7756		
Px_SP6	105388678	AGR92345.1	850	260	3.5909E-77	55.38		2.7790	−3.8940	
Px_SP7	105386078	AGR92347.1	894	262	1.1877E-57	50		2.4196		
Px_SP8	105393886	AGR92347.1	637	199	3.697E-108	98.97		−1.5772	−4.4863	1.1192
Px_SP9	105391896	AGR92347.1	633	199	4.768E-108	100			−1.0298	
Px_SP10	105388683	AGR92345.1	919	255	4.503E-140	94.12			−1.0937	
Px_SP11	105386077	AGR92347.1	891	264	9.174E-143	100			−1.3944	
Px_SP12	105391590	AGR92345.1	839	265	2.1485E-74	53.88			−1.7680	
Px_SP13	105391006	AGR92346.1	1,129	291	1.144E-109	73.53			−1.8202	
Px_SP14	105391005	AGR92346.1	974	292	1.594E-130	86.96			−2.3859	
Px_SP15	105391007	AGR92346.1	1,168	298	1.796E-121	74.32			−2.4715	
Px_SP16	105388679	AGR92345.1	820	258	1.9699E-85	59.69			−2.6043	−1.2320
Px_SP17	105386722	AGR92347.1	684	193	1.3776E-37	46.99				−1.8340
Px_SP18	105392197	ACR15995.1	2,022	269	1.6161E-55	41.95	1.2420			
Px_SP19	105390022	ACR15995.1	1,011	263	1.054E-47	39.74	1.1368		1.3599	−1.7433
Px_SP20	105380853	ADT80829.1	987	273	1.8982E-62	45.42	−1.5318	−3.4960	−2.1187	−3.1187
Px_SP21	105391955	ACR15993.2	871.8	241	8.7168E-26	34.21			−2.2016	
Px_SP22	105382233	ADT80828.1	1,954	609	9.247E-101	63.64			−1.6997	
Px_SP23	105389290	EHJ71121.1	5,328	1550	0	60.74			−3.2208	
Px_SP24	105392198	AGR92347.1	880	265	6.7877E-58	46.09				−1.2299
Px_SP25	105398563	XP_004929850.1	1,699	493	0	63.36		−1.3465	−2.6139	
Px_SP26	105380609	XP_004922188.1	1,544	416	6.376E-107	51.3			1.7734	
**Serine protease inhibitor**										
Serine Protease Inhibitor	105390805	EHJ65124.1	4,044	1003	0	54.85			1.0193	
**Serine proteinase**										
Px_SPN1	105384594	ACI45418.1	783.9	241	4.7416E-25	37.6			−1.6253	
Px_SPN2	105383822	AAQ22771.1	884	156	4.6358E-14	40.4			1.8963	
Px_SPN3	105394347	EHJ70457.1	1,615	450	2.5981E-82	41.12			1.1541	
Px_SPN4	105383519	NP_001040462.1	1,220	390	7.011E-132	60.31		−1.3974	1.3535	−2.0712
Px_SPN5	105395635	NP_001040462.1	769	244	2.1607E-35	60.16		−6.9542	1.2257	
Px_SPN6	105396174	AAR29602.1	1,874	484	1.6616E-83	51.49			1.7106	
**Trypsin-like serine protease**										
Px_Tryp_SP1	105381636	AAD21835.1	1,038	317	4.5535E-94	71.86	−4.3552	−9.2621	1.3827	−4.2621
Px_Tryp_SP2	105383595	ADK66277.1	728	225	3.7446E-55	46.64	1.0655			
Px_Tryp_SP3	105393197	EHJ67268.1	2,824	806	1.303E-101	52.21			−1.0000	
Px_Tryp_SP4	105380873	EHJ67268.1	2,612	805	3.687E-103	48.54			−1.7116	
Px_Tryp_SP5	105392836	AIR09766.1	696	156	2.696E-44	61.87		−1.5053	−2.6967	
Px_Tryp_SP6	105385090	AIR09766.1	872	156	3.2071E-44	61.87		−1.5560		
Px_Tryp_SP7	105394340	ACI32835.1	1,744	467	1.35E-148	65.95			1.1500	
Px_Tryp_SP8	105380637	ACI32835.1	1,705	464	1.891E-147	65.41			1.0114	
Px_Tryp_SP9	105392869	AIR09766.1	1,322	366	3.4186E-34	66.36			−1.6239	
**Trypsin-like serine protease**										
Px_Tryp_SPN1	105383936	ADK66277.1	1,277	271	7.1991E-50	42.63	−1.0741			
Px_Tryp_SPN2	105383572	ADK66277.1	902	271	2.0689E-49	46.75		1.7144		
Px_Tryp_SPN3	105385127	AEP25403.1	593	185	7.1148E-65	71.88		−3.7577	−1.5964	
Px_Tryp_SPN4	105387434	ADK66277.1	756	241	1.0998E-60	55.25		−4.6136	−2.3314	
Px_Tryp_SPN5	105383573	gb|ADK66277.1	1,020	270	2.1392E-48	42.7		2.9095	−4.5912	
Px_Tryp_SPN6	105392752	ADK66277.1	963	286	2.3853E-46	39.63			−2.8735	
Px_Tryp_SPN7	105383574	ADK66277.1	865	272	8.8986E-47	40			−2.8880	
Px_Tryp_SPN8	105383571	ADK66277.1	1,024	258	9.2395E-84	58.14			−3.6847	
Px_Tryp_SPN9	105387433	ADK66277.1	992	247	2.9458E-79	60.08			−4.1164	
Px_Tryp_SPN10	105386251	ADK66277.1	809	249	6.6173E-62	50.85			−10.300353	
Px_Tryp_SPN11	105386106	AEP25404.1	1,738	536	1.069E-129	92.13			−1.1830	
Px_Tryp_SPN12	105393249	AFK93534.1	1,904	490	1.017E-120	50.75	1.0059		3.0422	
Px_Tryp_SPN13	105397224	AFK93534.1	1,673	290	3.867E-121	51.01		1.5802	3.9802	
Px_Tryp_SPN14	105386282	AFK93534.1	2,100	657	2.7677E-82	50.18			3.9580	−1.1229
Px_Tryp_SPN15	105391595	AFK93534.1	1,629	485	1.285E-137	50.72			1.9038	
**Chymotrypsin like serine protease**										
Px_ChymTryp_SP1	105388850	EHJ70525.1	944	300	6.2658E-52	44.84			−3.8146	
Px_ChymTryp_SP2	105381896	AFM77773.1	973	249	5.0365E-76	56.41		1.6944		
Px_ChymTryp_SP3	105380855	AFM77775.1	944	282	2.877E-89	57.8		−1.1103	−1.8191	
Px_ChymTryp_SP4	105388849	NP_001040430.1	1,147	304	3.1128E-60	47.08			−3.2694	
Px_ChymTryp_SP5	105394289	AIR09764.1	1,054	300	7.4974E-52	43.32			−3.4467	1.0378
Px_ChymTryp_SP6	105397690	ACI45417.1|	318	91	4.39E-18	48.91			2.5571	
Px_ChymTryp_SP7	105383260	NP_001040178.1	939	289	8.9544E-67	47.81	2.2236			
**Kazal-type inhibitor**										
Px_KTI1	105382984	ADF97836.1	802	190	1.5693E-23	37.72				−1.1667
**Serpin**										
Px_Serpin1	105396587	BAF36821.1	1,659	450	0	99.33			1.1162	
Px_Serpin2	105387806	BAF36820.1	1,262	394	0	99.75			−1.1952	−1.3669
Px_Serpin3	105392292	dbj|BAF36820.1	601	199	5.9941E-06	55.81			−1.7840	−2.4646
Px_Serpin4	105392280	BAF36820.1	1,321	400	0	97				−1.4842
Px_Serpin5	105383392	BAM18904.1	1,931	510	0	66.23	−4.5814		−1.3755	−3.1685
Px_Serpin6	105387001	AEW46892.1	1,523	413	9.804E-169	72.17	−1.4818		1.6229	
Px_Serpin7	105390554	AEW46895.1	1,742	398	8.829E-108	48.26	−1.5187			
Px_Serpin8	105383829	NP_001037021.1	445	138	3.1274E-32	46.58	−1.5259	−1.3060		
Px_Serpin9	105398773	EHJ65045.1	2,173	607	2.5594E-38	55.78		1.5502	1.5146	−1.4854
Px_Serpin10	105381092	EHJ65951.1	2,169	651	1.2911E-90	71.37			−1.2257	
Px_Serpin11	105386098	ACG61190.1	5,485	1418	0	54.61			−1.6450	
Px_Serpin12	105390552	NP_001037205.1	1,683	397	3.282E-136	60.2	−1.0957			
Px_Serpin13	105383513	NP_001139702.1	2,683	387	5.0332E-63	36.75	−1.6280	−5.0875	1.3388	
Px_Serpin14	105389206	NP_001139706.1	1,763	407	2.4774E-57	34.28		1.3641		
Px_Serpin15	105387669	NP_001139701.1	1,391	401	2.0403E-93	46.21				−1.0807
**SIGNALLING PATHWAY**										
Px_Myd88	105393101	AFK24444.1	1,305	381	8.633E-107	52.16			2.2204	
Px_Spatzle	105385965	NP_001243947.1	1,797	418	2.561E-142	59.08			−2.5386	
**EFFECTORS**										
**Prophenoloxidase**										
Px_PPO1	105393828	BAF36824.1	1,558	405	0	92.58		−1.5230	2.7326	
Px_PPO2	105393465	BAF36824.1	2,479	790	1.822E-144	92.28			2.1137	
**Moricin**										
Px_Moricin1	105392531	ABQ42576.1	434	65	1.9938E-10	76.32			7.2646	−7.9307
Px_Moricin2	105392533	ABQ42576.1	436	65	1.0544E-11	75	−1.9629	−2.1231	3.9596	−3.3033
Px_Moricin3	105392532	ABQ42576.1	451	65	2.0342e-10/	76.32	−9.5793	−2.4708	5.5358	−1.8311
**Cecropin**										
Px_Cecropin1	105394859	ADA13281.1	684	65	1.5836E-17	73.85	−2.5093	−6.0395		−4.6154
Px_Cecropin2	105397888	ADA13281.1	582	65	1.0647E-17	73.85	−3.4452	−6.0700		−3.9365
Px_Cecropin3	105394858	ADA13281.1	512	65	2.0599E-17	73.85		−4.5206		−3.2365
Px_Cecropin4	105392561	ADR51147.1	398	61	1.2033E-15	65.08	−5.2695	−5.1013		
Px_Cecropin5	105394860	BAF36816.1	510	65	1.0252E-16	73.02	−1.9265	−5.0688		
**Gloverin**										
Px_Gloverin1	105389810	ACM69342.1	628	172	5.0444E-54	60.57	−1.2012	−4.8084		
Px_Gloverin2	105389803	ACM69342.1	489	128	1.9253E-51	89.91	−1.3116	−4.8361		−2.6256
**Lysozyme**										
Px_Lys1	105382813	EHJ67777.1	548	140	6.7928E-50	71.54		−1.3225		
Px_Lys2	105381977	NP_001093293.1	1,345	143	1.8353E-51	75.63	−10.871135	−3.2201	−1.9733	−4.2418
**OTHERS**										
**Peroxidase**										
Px_Peroxidase1	105382493	XP_004924228.1	2,008	640	3.621E-124	39.14			1.1018	
Px_Peroxidase2	105388497	BAM17900.1	2,079	627	1.319E-177	50.66	1.9139	−1.2812	−2.7023	2.2984
Px_Peroxidase3	105389833	EHJ67854.1	824	271	8.222E-132	82.02	−7.6724	−1.5227	1.1856	
Px_Peroxidase4	105390475	EHJ75729.1	2,917	753	0	67.72		1.0000		
Px_Peroxidase5	105396491	BAM17900.1	1,614	537	4.194E-157	51.61		−2.6129	−1.6793	2.1894
Px_Peroxidase6	105394585	EHJ75729.1	2,218	548	0	73.16			−1.3796	
**Integrin**										
Px_Integrin1	105383688	ABF59518.1	630	176	3.8043E-26	57.14	2.5850		2.9336	2.7137
Px_Integrin2	105383715	ABF59518.1	992	290	1.1377E-22	28.99	−1.0139		−1.0806	
Px_Integrin3	105392513	ABF59518.1	1,922	639	5.9709E-44	27.22		−1.4097	1.3976	
Px_Integrin4	105386410	EHJ72232.1	627	172	1.3367E-30	48.3				1.3943
Px_Integrin5	105387843	EHJ72232.1	2,713	876	0	50.79			1.4047	1.2135
Px_Integrin6	105394193	ACS66819.1	2,349	746	0	90.3	−1.0118			−1.2063
Px_Integrin7	105393654	AAO85804.1	1,669	556	0	69.45	−1.1524			−1.1137
Px_Integrin8	105380096	AII79417.1	2,240	543	3.284E-113	69.72	−1.0752		−1.5091	
**Transferrin**										
Px_Transferrin1	105393952	dbj|BAF36818.1	1,006	325	0	99.05		−2.6292	2.0508	−1.2249
Px_Transferrin2	105384728	BAF36818.1	1,904	534	0	96.89		−2.7590	1.3416	−1.3851
**Thioredoxin**										
Px_Thioredoxin1	105380321	AHK05704.1	1,232	247	6.452E-125	87.45		−1.3545	−1.0976	
Px_Thioredoxin2	105398803	XP_004925107.1	1,861	266	2.975E-117	77.73		−2.1099		
**Catalase**										
Px_Catalase1	105398438	NP_001036912.1	1,767	508	0	82.09			1.3045	−1.6592
Px_Catalase2	105390515	NP_001036912.1	1,686	508	0	82.48				−1.4120
Px_Catalase3	105389213	XP_004924808.1	1,429	474	1.83E-145	53.4	1.3567		−3.6721	
Px_Catalase4	105385727	XP_004924808.1	1,676	530	2.181E-148	52.87	1.2412		−3.1595	
**Hemolin**										
Px_Hemolin1	105394779	ACN69054.1	1,451	415	0	94.46	−1.3656	−2.2910		−1.9475
Px_Hemolin2	105382056	ACN69054.1	1,403	415	0	94.46		−2.7738		−1.4243
**Oxidase**										
Px_Oxidase	105390649	BAM20596.1	3,273	1032	0	84.16			3.1726	−1.6638

The entomopathogenic fungi are recognized as an environmentally friendly tactic for controlling the insect pests. Previously, the entomopathogenic fungi like *M. anisopliae* and *B. bassiana* have received an increasing attention due to wide host range and capability of mass production (Butt et al., 2001). Recently, it has been shown that *I. fumosorosea* also has the potential to control various insect pests (Gökçe and Er, 2005; Huang et al., 2010; Avery et al., 2011). Therefore, considering the importance of *I. fumosorosea*, a genomic analysis of immune response of *P. xylostella* following infection of *I. fumosorosea* at different time points using high-throughput sequencing Illumina was performed in the present study.

#### Immune recognition families

**Figure 6:**
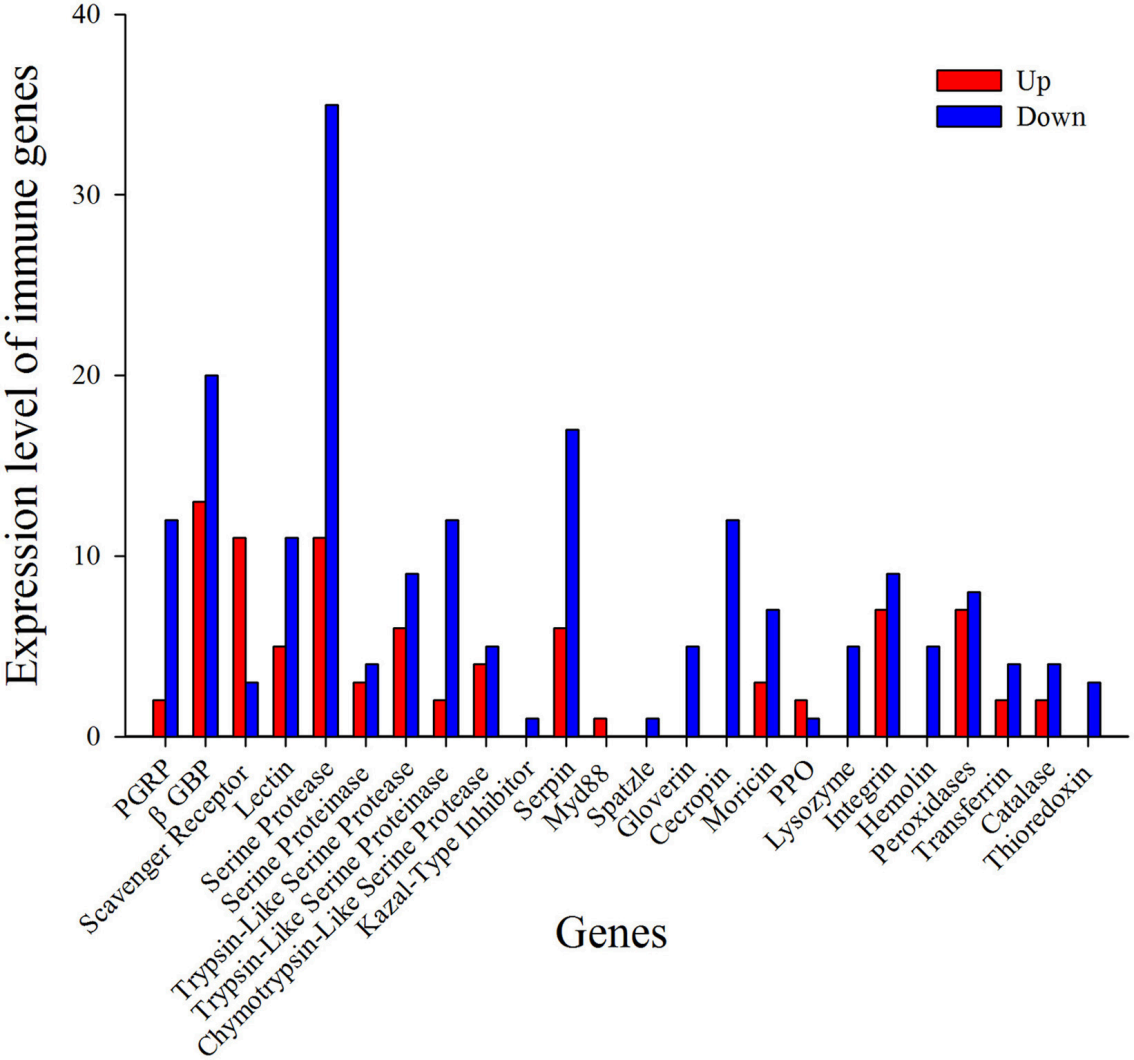
Functional classification of immunity- related DEGs in response to *I. fumosorosea*.

Recognition of pathogen is the initial step in the defense against invading microbes, eliciting cellular and humoral responses. Pathogens produce conserved pathogen-associated molecular patterns (PAMPs) and the host produces pattern-recognition receptors (PRRs) in response (Mogensen, 2009). PRRs like peptidoglycan recognition proteins (PGRPs), β -Glucan binding proteins (GNBPs), lectins, scavenger receptors, and hemolin bind to the PAMPs (Hultmark, 2003). Insect PGRPs can trigger signal transduction through the Toll pathway, leading to the activation of AMP production (Zaidman-Rémy et al., 2011). In the present report, 14 PGRPs were identified and most of them were down-regulated after treatment with *I. fumosorosea* (Figure 6 and Table 3). Among the down-regulated PGRPs, two PGRP transcripts (px_105387866 and px_105386207) were down-regulated up to 2-fold (−2.60 and −2.21), respectively at 12 h post-treatment. Previously, it has also been shown that PGRPs were down-regulated after the injection of secondary metabolite (destruxin) of *M. anisopliae* in *D. melanogaster* (Pal et al., 2007), whereas in contrast, PGRPs were up-regulated in response to *M. acridium* and *Beauveria bassiana* in *Helicoverpa armigera* and *Ostrinia furnacallis* (Liu et al., 2014; Xiong et al., 2015). The expression of other PRRs like lectins, hemolin, and GNBPs was also down-regulated after treatment with *I. fumosorosea* (Figure 6 and Table 3). Among PRRs, only the scavenger receptors were up-regulated at all-time points post-infection. Our results are in accordance with a previous report showing that among PRRs, only scavenger receptors were up-regulated in response to destruxin A in *D. melanogaster* (Pal et al., 2007). Our results suggest that PRRs like PGRPs, GNBPs, lectins, and hemolin may be the target of *I. fumosorosea* and scavenger receptors are responsible for the activation of the immune response of *P. xylostella* to *I. fumosorosea*.

#### Toll and imd signaling pathways

The Toll pathway is primarily activated by fungi and Gram-positive bacteria while the Gram-negative bacteria triggers the activation of Imd pathway leading to the production of AMPs (Aggarwal and Silverman, 2008; Hetru and Hoffmann, 2009). Here, in our study, we found that only spatzle and MyD88 showed differential expression while the other immune genes of toll pathway were not induced after treatment with *I. fumosorosea* (Figure 6 and Table 3). Of note, Imd pathway was also not induced after the treatment with *I. fumosorosea* at different time points. The expression of MyD88 was up-regulated whereas, spatzle showed down-regulated expression after treatment (Figure 6 and Table 3). Previously, a similar phenomenon was observed in *D. melanogaster* where only pelle and toll showed differential expression in the Toll pathway, and Imd pathway was not induced after injection of destruxin A (Pal et al., 2007). Thus, our results show that *I. fumosorosea* has the ability to suppress the expression of toll pathway genes and in the meantime *P. xylostella* could resist the infection of *I. fumosorosea*.

#### Melanization

Melanization is considered as a vital component of the immune system of insects. It regulates the melanin cascade mediated by prophenoloxidases (PPO) (Taft et al., 2001). When a pathogen invades, PPO gets activated and transformed into PO following transformation of phenolic substances into quinone intermediates and ultimately killing pathogens. Here, only three PPO were found after the treatment of *P. xylostella* with *I. fumosorosea* and two of them were up-regulated up to 2-fold at 12 h post-infection.

Serine proteases represent a very large group of enzymes in almost all organisms and are involved in various biological processes (Ross et al., 2003). The structure of serine proteases consists of His, Asp, and Ser amino acid residues forming a catalytic triad (Perona and Craik, 1995). Generally, serine proteases are inactive pro-enzymes and need proteolytic cleavage for activation (Ross et al., 2003). Notably, many serine proteases identified in our study showed up- and down-regulated expression with a serine protease (px_105393891) showing highly up-regulated expression (10.77) and a serine protease (px_105381636) showing down-regulated expression (−9.26) after treatment with *I. fumosorosea* at 18 h post-infection (Figure 6 and Table 3). It has been reported that the serine proteases showed same up- and down-regulated expression in *P. xylostella* and *D. melanogaster* after treatment with destruxin A (Pal et al., 2007; Han et al., 2013).

Serpins, a super-family of proteins, are found in nearly all organisms (Gettins, 2002). In general, they contain 350–400 amino acid residues. The similarity of amino acid sequence ranges from 17 to 95% among all serpins. They contain three β-sheets and seven to nine α-helices folding into a conserved tertiary structure with a reactive center loop (RCL) (Gettins, 2002). The RCL of these serpins binds to the specific active site of the target proteinase. When the cleavage of the serpin takes place at scissile bond, it goes through an important conformational change, trapping the target proteinase covalently (Dissanayake et al., 2006; Ulvila et al., 2011). Interestingly, almost all the serpins were down-regulated at an early stage of treatment at 12 h post-infection. In contrast, the expression level of serpins was up-regulated in *P. xylostella* after treatment with *Diadegma semiclausum* parasitization (Etebari et al., 2011; Han et al., 2013). The activation of serpins by *D*. *semiclausum* in previous reports may be a strategy to suppress the activity of PPO in the host defense system.

#### Antimicrobial peptides

AMPs are evolutionarily conserved low molecular weight proteins and play a vital part in the insect defense system against microorganisms (Bulet and Stocklin, 2005). Here, in the present study, lysozyme, moricin, gloverin, and cecropin were identified after the treatment of *P. xylostella* with *I. fumosorosea* at different time periods. Interestingly, all the AMPs were down-regulated after treatment with *I. fumosorosea* (Figure 6 and Table 3) The expression of lysozyme (px_105381977) was decreased up to 10-fold (−10. 87) at 12 h post-infection, moricin (px_105392532) expression was decreased up to 9-fold (−9.57) at 12 h post-infection, gloverin (px_105389810) expression was reduced up to 4-fold (−4.80) at 18 h post-infection, and the expression of cecropin (px_105394859) was down-regulated up to 6-fold (−6.03) at 18 h post-infection of *I. fumosorosea*. Previously, most of the reports on immune response of insects to entomopathogenic fungi identified that the expression of AMPs was up-regulated after the treatment leading to a conclusion that the entomopathogenic fungi were unable to suppress the immune system (Liu et al., 2014; Xiong et al., 2015; Zhang et al., 2015). However, varroa mites and destruxin A were reported to suppress the expression of AMPs in *Apis mellifera* and *D. melanogaster* (Gregory et al., 2005; Pal et al., 2007). The immune response suppression in host by an entomopathogenic fungi such as, *I. fumosorosea* would have obvious benefits for success of pathogenic fungi. Previously, it was observed that when mutations were introduced in Toll and IMD pathways, the *D. melanogaster* was unable to produce AMPs resulting in extreme vulnerability to fungal challenge (Lemaitre et al., 1996; Tzou et al., 2002). Thus, the ability to reduce AMP production is likely to aid fungal survival in a variety of insect hosts. A similar suppression of AMPs in our study by *I. fumosorosea* adds a new dimension to the dynamics of host-pathogen interactions.

## Conclusion

Concluding our findings, the present study adopted genomic analysis with RNA-Seq and DGE technology to find out DEGs especially focusing on immunity-related DEGs after treatment with *I. fumosorosea*. It is speculated that the entomopathogenic fungi *I. fumosorosea* not only down-regulated the expression of PRRs and other immune genes but also the activity of AMPs was inhibited leading to the ultimate suppression of the immune system of *P. xylostella*. Thus, it shows that *I. fumosorosea* has the potential to suppress the immune system of *P. xylostella* and can be adopted as a bio-pesticide for *P. xylostella* control. Our study explores a new avenue in research to develop bio-pesticides for controlling *P. xylostella* by focusing on the insect immune system.

## Ethics statement

Our work confirms to the legal requirements of the country in which it was carried out.

## Author contributions

Conceived and designed the experiments: FJ, MS, XiaoxX, and JX. Performed the experiments: JX, XiaoxX, and MS. Analyzed the data: XiaojX, JY, XZ, and JX. Contributed reagents/materials/analysis tools: SL and SW. Wrote the manuscript: MS, XiaoxX, and JX. Revised the manuscript: MS, FJ, and XY.

### Conflict of interest statement

The authors declare that the research was conducted in the absence of any commercial or financial relationships that could be construed as a potential conflict of interest.
